# Detection of Specific Immunoglobulins in the Saliva of Patients With Mild COVID-19

**DOI:** 10.7759/cureus.52113

**Published:** 2024-01-11

**Authors:** Sara Akeel, Soulafa Almazrooa, Ahoud Jazzar, Amal Mohammed Sindi, Nada J Farsi, Nada Binmadi, Reem Badkok, Modi Aljohani, Sarah AlFarabi

**Affiliations:** 1 Department of Oral Diagnostic Sciences, Faculty of Dentistry, King Abdulaziz University, Jeddah, SAU; 2 Department of Dental Public Health, Faculty of Dentistry, King Abdulaziz University, Jeddah, SAU; 3 Faculty of Dentistry, King Abdulaziz University, Jeddah, SAU

**Keywords:** serum immunoglobulin a, oral immunoglobulin, enzyme linked immunosorbent assay (elisa), saliva testing, sars-cov-2 and covid-19

## Abstract

Saliva has many advantages over blood as a biofluid, so using it for measuring and monitoring antibody responses in COVID-19 would be highly valuable. To assess the value of saliva-based IgG and IgM/IgA antibody testing in COVID-19, this cross-sectional pilot study evaluated the accuracy of salivary and serum IgG and IgM/IgA for detecting mild COVID-19 and their correlation. Fifty-one patients with mild COVID-19 (14-28 days post-symptom onset) were included in the study. Enzyme-linked immunosorbent assays (ELISA) were used to measure IgG and IgM/IgA responses to SARS-CoV-2 spike protein in both serum and saliva samples using a slightly modified protocol for saliva samples. Saliva-based IgG testing had 30% sensitivity and 100% specificity, with a positive predictive value (PPV) of 100% and a negative predictive value (NPV) of 50%. Saliva-based IgM/IgA testing had 13.2% sensitivity and 100% specificity, with a PPV of 100% and an NPV of 28.3%. Blood and saliva IgG values were positively correlated. Saliva currently has limited diagnostic value for COVID-19 testing, at least for mild disease. Nevertheless, the significant positive correlation between blood and saliva IgG titers indicates that saliva might be a complementary biofluid for assessing systemic antibody responses to the virus, especially if the assay is further optimized across the full disease spectrum.

## Introduction

The COVID-19 pandemic caused widespread global disruption, the SARS-CoV-2 virus continues to circulate, and the threat of new variants persists. COVID-19 is diagnosed and monitored clinically by chest CT and in the laboratory by reverse transcription polymerase chain reaction (RT-PCR)-based RNA detection of SARS-CoV-2 in oropharyngeal or nasopharyngeal swabs and by enzyme-linked immunosorbent assay (ELISA)-based antibody detection in serum samples [1].

The detection of specific antibodies targeting SARS-CoV-2 in serum can be used to both diagnose and monitor COVID-19. However, applying blood-based assays has several drawbacks, including patient discomfort, the need for qualified healthcare workers to take blood samples, and the risk of microbial transmission [2]. Hence, there is a need for sensitive, accurate, rapid, and inexpensive tests to screen infected individuals and monitor their immune responses.

Saliva is a biofluid that overcomes many of the disadvantages of using blood for antibody testing. It can easily be collected by the patient, minimizing the exposure of healthcare workers to biological samples and reducing the need for trained workers, as well as simplifying sample collection and transport and reducing costs. A simple, low-cost saliva-based assay could facilitate population sampling and epidemiological studies. Saliva contains many proteins, including immunoglobulins, hormones, and enzymes, but these biomolecules are often present at much lower concentrations than in serum, necessitating highly sensitive assays [3]. Nevertheless, saliva-based viral assays have been developed as non-invasive, simple, and inexpensive tools for detecting immunoglobulins against HIV, human papillomavirus, herpes simplex [4], Zika [5], measles, mumps, and rubella viruses [6].

While there have been many studies of immune responses to COVID-19 in serum, plasma, and blood samples, only a few studies have examined the value of saliva as a suitable, accurate, and sensitive biofluid for the detection of anti-SARS-CoV-2 antibodies. To et al. used saliva samples from the posterior oropharynx and serum specimens from 23 COVID-19 patients to monitor viral load [7], noting a high viral load in saliva and an increase in serum IgG and IgM antibody levels in 43% of patients ten days or later after symptom onset. Directly testing antibody levels in saliva, Randad et al. demonstrated that SARS-CoV-2 antigen-specific antibody responses in saliva reflect those observed in serum using a multiplex SARS-CoV-2 antibody immunoassay [8]. Due to the variability observed in these findings, there is an interest in investigating alternative and cost-effective methods for monitoring COVID-19.

Here, to explore the value of saliva antibody testing in patients with COVID-19, we investigated the suitability of salivary specimens for detecting IgM and IgG/IgA antibodies specific to SARS-CoV-2 in a population of COVID-19 patients with mild disease in Jeddah, Saudi Arabia, and explored associations between salivary and serum antibody levels and clinical symptoms during infection.

## Materials and methods

This was a cross-sectional study in which samples of human serum and saliva were collected from individuals with mild symptoms who tested positive for SARS-CoV-2 through RT-PCR, as well as from healthy, age- and sex-matched controls who had not been previously infected. These specimens were acquired from individuals attending the university hospital in Jeddah, Saudi Arabia, during the period from November 2020 to February 2021. The King Abdulaziz University Research Ethics Committee (107-11-20) approved the study, and all participants provided written, informed consent prior to their enrollment.

Participant enrollment and consent

Demographic and medical data were collected from patients with the first infection with mild COVID-19 defined according to WHO criteria for COVID-19 severity (WHO, 2020). All infected participants were unvaccinated adults testing positive for SARS-CoV-2 by RT-PCR analysis of nasopharyngeal or oropharyngeal specimens 14 to 28 days after symptom onset. Patients under 18 years of age, those with a medical history of chronic disease and on immunosuppressive medications, and those with a history of MERS-CoV infection were excluded. Control serum and saliva samples were collected from 19 asymptomatic individuals who had never tested positive for SARS-CoV-2 by RT-PCR and had not been vaccinated.

A checklist of all possible symptoms associated with infection was provided to participants to record their symptoms over the first two weeks of infection (fever, chills, cough, shortness of breath, difficulty breathing, fatigue, muscle or body aches, headache, loss of taste or smell, sore throat, congestion or runny nose, nausea or vomiting, diarrhea, pain or pressure in the chest, confusion, inability to wake or stay awake, and pale, gray, or blue-colored skin, lips, or nail beds). Oral lesions during infection, such as oral ulcers, gingival inflammation, gingival enlargement, and burning sensations, were also self-reported by participants.

Specimen collection and processing

Blood samples were collected by laboratory technicians. Saliva samples were collected and supervised by a trained provider, as previously described [9]. Participants were asked to refrain from eating, drinking, smoking, or using oral hygiene products for one hour before collection. They were asked to spit their saliva into a sterile plastic tube. The provider wrote the participant’s name, file number, and date of collection on a cold container before the specimen was transferred to the King Abdulaziz University Faculty of Dentistry laboratory. Blood samples were centrifuged at 5000 rpm for 10 minutes, and saliva samples were centrifuged at 3000 rpm for 5 minutes. The supernatant was then immediately transferred and aliquoted (0.5 mL) into small Eppendorf tubes and stored at -80°C.

Serology

SARS-CoV-2 ELISA IgG (Vircell, Granada, Spain) and SARS-CoV-2 ELISA IgM/IgA (Vircell) targeting the spike protein were performed according to the manufacturer’s protocol modified for salivary sample dilutions based on a similar study (serum 1:20, saliva 1:5 dilution) [10]. The lower dilution for saliva samples was the only modification to the protocol. All samples were run in duplicate. Absorbance was measured immediately at 450 nm and 630 nm, and the 630 nm optical density (OD) was subtracted from the 450 nm OD. Data were analyzed as recommended by the manufacturer, and the results were reported as a ratio according to the equation for the Ab index: (sample OD/cut-off serum or saliva mean OD)*10. The positive and negative controls acted as a calibrator, which was used to normalize antibody signals for cross-plate comparison.

Statistical analysis

The diagnostic accuracy of salivary IgG and IgM/IgA for detecting COVID-19 infection was assessed by comparing the results with gold standard blood IgG and blood IgM/IgA in the same patients. Demographic variables and symptoms are described as counts and percentages for categorical variables and as means and standard deviations for continuous variables.

Participants were categorized as follows: “true positive” (TP) if diagnosed with COVID-19 by both saliva and blood; “true negative” (TN) if negative for COVID-19 by both test modalities; “false positive” (FP) if positive in the salivary test but not the blood test; and “false negative” (FN) if diagnosed by the blood test but not the salivary tests. The sensitivity, specificity, positive predictive value (PPV), negative predictive value (NPV), kappa, and agreement percentage were calculated according to the formulas: sensitivity = TP / (TP + FN); specificity = TN / (TN + FP); PPV = TP / (TP + FP); NPV = TN / (TN + FN); and agreement percentage = total number of agreements / total number.

IgG and IgM/IgA ODs were not normally distributed according to inspection of histograms and quantile-quantile plots and the results of skewness and kurtosis tests for normality. Therefore, non-parametric tests were used. The mean ODs of blood and saliva IgGs and IgM/IgA were compared using Wilcoxon rank sum tests. Correlations were assessed by calculating the corresponding Spearman correlations. The Pearson correlation coefficient was calculated to assess the correlation between serum and saliva IgG and IgM/IgA values with a 95% confidence interval (CI).

Statistical analyses were performed using Stata v12.1 software (StataCorp LP, College Station, TX, USA). A P-value <0.05 was considered significant.

## Results

Fifty-one patients who tested positive for SARS-CoV-2 by RT-PCR of nasopharyngeal and/or oropharyngeal swab samples and 18 healthy, age- and sex-matched controls who had not been previously infected were included in the study. The mean age of the patients was 28 ± 7.7 years, and 61% of the patients were male. The most common symptoms reported were fever (61%) and headache (41%). A summary of demographics, symptoms, and ELISA results for anti-SARS-CoV-2 IgG and IgM/IgA antibodies in both serum and saliva is presented in Table 1 for COVID-19-positive patients.

**Table 1:** Characteristics of patients with mild COVID-19

Variables	N (%)
Demographic factors and COVID-19 antibodies	
Age, mean (SD)	27.6 (7.7)
Sex	
Male	31 (60.8)
Female	20 (39.2)
Current smoker	
No	32 (62.8)
Yes	19 (37.3)
COVID-19 antibodies	
IgG in blood, mean (SD)	0.78 (0.7)
IgG in saliva, mean (SD)	0.22 (0.2)
IgM/IgA in blood, mean (SD)	0.80 (0.7)
IgM/IgA in saliva, mean (SD)	0.19 (0.2)
Symptoms	
Oral lesions	
No	42 (82.4)
Yes	9 (17.7)
Headache	
No	30 (58.8)
Yes	21 (41.2)
Fever	
No	20 (39.2)
Yes	31 (60.8)
Cough	
No	34 (66.7)
Yes	17 (33.3)
Common cold	
No	45 (88.2)
Yes	6 (11.8)
Ageusia	
No	39 (76.5)
Yes	12 (23.5)
Tiredness	
No	50 (98.0)
Yes	1 (2.0)
Anosmia	
No	32 (62.8)
Yes	19 (37.3)
Difficulty breathing	
No	45 (88.2)
Yes	6 (11.8)
Diarrhea	
No	49 (96.1)
Yes	2 (3.9)
Chills	
No	49 (96.1)
Yes	2 (3.9)
Chest pain	
No	50 (98.0)
Yes	1 (2.0)
Fatigue	
No	40 (78.4)
Yes	11 (21.6)
Nausea	
No	47 (92.2)
Yes	4 (7.8)
Runny nose	
No	42 (82.4)
Yes	9 (17.7)

The diagnostic accuracy of salivary IgG for diagnosing COVID-19 was assessed, as shown in Tables 2-3. All patients who were negative for blood IgG were also negative for salivary IgG (TN). However, of 30 patients positive for blood IgG, only nine were positive for salivary IgG (30%) (TP). The sensitivity was 30% (95% CI 14.7-49.4), and the specificity was 100% (95% CI 83.9-100).

**Table 2:** Positive predictive value (PPV) and negative predictive value (NPV) of anti-SARS-CoV-2 IgG antibodies in saliva

		IgG in blood		Total
		Present	Not present	
IgG in saliva	Present	9 true positive (TP)	0 false positive (FP)	9
	Not present	21 false negative (FN)	21 true negative (TN)	42
Total		30	21	51

**Table 3:** The diagnostic accuracy of salivary IgG for diagnosing COVID-19

Disease prevalence (95% CI)	59 (44-72.4)
Sensitivity (95% CI)	30 (14.7-49.4)
Specificity (95% CI)	100 (83.9-100)
Positive predictive value (PPV) (95% CI)	100 (66.4-100)
Negative predictive value (NPV) (95% CI)	50 (34.2-65.8)
Kappa (%)	26.1 (9.8-42.4)
Agreement (%)	58.82

The diagnostic accuracy metrics of IgM/IgG for detecting COVID-19 are shown in Tables 4-5. There were 13 TN cases and no FP cases. However, of the 38 cases positive for blood IgM/IgA, only five were TP, and 33 were FN. The sensitivity was 13.2% (95% CI 4-41-28.1), and the specificity was 100% (95% CI 75.3-100).

**Table 4:** Positive predictive value (PPV) and negative predictive value (NPV) of anti-SARS-CoV-2 IgM/IgA antibodies in saliva

		IgM/IgA in blood		Total
		Present	Not present	
IgM/IgA in saliva	Present	5 true positive (TP)	0 false positive (FP)	5
	Not present	33 false negative (FN)	13 true negative (TN)	46
Total		38	13	51

**Table 5:** The diagnostic accuracy of salivary IgM/IgA for diagnosing COVID-19

Disease prevalence (95% CI)	75 (60-85.7)
Sensitivity (95% CI)	13.2 (4.41-28.1)
Specificity (95% CI)	100 (75.3-100)
Positive predictive value (PPV) (95% CI)	100 (47.8-100)
Negative predictive value (NPV) (95% CI)	28.3 (16-43.5)
Kappa (%)	7.2 (0.2-14.2)
Agreement (%)	35.29

There was a statistically significant difference between mean IgG and IgM/IgA levels detected in the blood and saliva, with higher levels in the former (P<0.001; Table 6). Blood and saliva IgG were positively correlated (Spearman’s rho 0.302, P=0.031), but blood and saliva IgM/IgA were not positively correlated (Table 6). None of the clinical symptoms were associated with blood IgM/IgA except oral lesions, where 90% of IgM/IgA-positive patients had oral lesions compared with 62% of IgM/IgA-negative patients (P=0.036; data not shown).

**Table 6:** Comparison of mean optical densities of IgG and IgM/IgA in blood and saliva, and the correlations between blood and saliva values ^Wilcoxon rank sum test, *Spearman correlation

	IgG				P-value^	IgM/IgA				P-value^
	Blood		Saliva			Blood		Saliva		
	Mean (SD)	Median	Mean (SD)	Median		Mean (SD)	Median	Mean (SD)	Median	
	0.78 (0.7)	0.49	0.22 (0.2)	0.17	<0.001	0.80 (0.7)	0.48	0.19 (0.2)	0.15	<0.001
Correlation*	0.302				0.031	0.038				0.791

All control samples were negative for all tested antibodies. The patients were all healthy with no reported medical issues, but 19% reported smoking and 39% reported loss of taste.

## Discussion

There is a need to develop and validate simple, cheap, and non-invasive methods to monitor SARS-CoV-2 infection status and immune responses. From a public health perspective, having options for patient-collected samples could facilitate population-based studies to measure the population prevalence of current and past infection with SARS-CoV-2. Such studies are critical for understanding the natural history of infection, developing an understanding of what proportion of the population has asymptomatic infection, and monitoring population immunity [11]. Furthermore, saliva samples have the reported benefits of reduced time and cost of handling compared with other specimen types [7]. Several studies have compared the viral load (titer) sensitivity (positivity) in saliva with their paired nasopharyngeal swabs by RT-PCR, finding that saliva is an equally effective biofluid for detecting COVID-19 [12-15]. However, other published studies have examined the diagnostic accuracy of salivary antigen tests in real-life settings and observed lower values than those reported by the manufacturer, with an overall sensitivity ranging from 12% to 67% [16-19].

The aim of the present cross-sectional study was to evaluate the effectiveness of saliva as a diagnostic biofluid to detect immunoglobulins targeting SARS-CoV-2 in COVID-19 patients. The commercially available plates all cater to serum diagnosis, but we hypothesized that these plates could also be used to detect salivary antibodies to SARS-CoV-2.

Our detection of antibodies in the first month after infection in both the serum and saliva is similar to previously published studies [10]. COVID-19-specific IgG antibodies were detected with 100% specificity and 30% sensitivity. There have been other previous attempts to detect salivary antibodies, with variable success. MacMullan et al. optimized various elements of saliva COVID-19 testing by ELISA, including sample handling and collection, sample dilution, sample preparation, and incubation periods [20]. They optimized the protocol by specifically decreasing sample dilution and adding blocking buffer, achieving a sensitivity of 84%. However, the timeframe for salivary sample collection that reached this sensitivity was not identified by the authors; it extended to any period beyond 21 days post-symptom onset. Interestingly, this study shows that the sensitivity to detect antibodies against SARS-CoV-2 in saliva samples increases with age, especially above 40 [20]. Another study demonstrated the detection of anti-SARS-CoV-2 antibodies by ELISA in the saliva of seropositive individuals [21], with a sensitivity and specificity of >90% for the detection of SARS-CoV-2-specific IgG antibodies against one of the three Cys-like protease protein targets tested. This study also observed a trend for higher antibody responses in patients with more severe disease and older individuals, consistent with other studies [20-22]. This may explain the low sensitivity in our results, considering that the mean age in our study is 28, and we only included healthy individuals with mild cases of the disease. Our study revealed that anti-SARS-CoV-2 IgG antibodies were detectable in 59% of serum and 18% of saliva samples of infected individuals and were not present in any of the healthy controls. All cases that were positive in saliva were also positive in serum, suggesting that salivary IgG is of systemic origin. IgM/IgA anti-SARS-CoV-2 antibodies were found in 76% of serum and 10% of saliva samples, with a combined positivity of 76% and 20% in serum and saliva, respectively. IgM antibodies were therefore more readily detected than IgG antibodies. IgM antibodies are produced as the host’s first response to a new infection, providing short-term protection, increasing for several weeks, and then declining as IgG production begins. Accordingly, IgM antibodies are readily detectable at 21 days post-infection [10]. Indeed, another study reported 100% sensitivity of salivary anti-NP IgG and 100% specificity of anti-RBD IgG, with their antibody responses corresponding to those seen in serum [23]. However, this study lacked consistent information regarding the severity of COVID-19 cases, which could have influenced the quality and kinetics of antibody responses. Furthermore, the absence of paired specimens in both serum and saliva assays from the same subjects prevented a direct comparison.

We only adjusted the sample dilution without altering any other of the manufacturer’s instructions. Optimization studies have generally found high background noise, especially against IgA, which ultimately affects assay specificity [10], necessitating further optimizations for salivary IgA anti-SARS-Cov-2 antibodies. Additionally, it would be useful to specify whether the detected IgA was derived from serum or whether it was secretory IgA (s-IgA) since the virus affects mucosal sites (i.e., respiratory, nasal, and oral) to produce s-IgA, which might affect assay interpretation.

We detected a positive correlation between anti-SARS-CoV-2 IgG antibodies in the serum and saliva, confirming that saliva accurately reflects the systemic immune status. Although the results signified a positive correlation between IgG and IgM in serum, it would be interesting to measure the dynamics of these changes over time. The body retains a catalogue of IgG antibodies that can be rapidly reproduced upon exposure to the same antigen. In this way, IgG antibodies form the basis of long-term protection against disease, as exploited by vaccination-induced immunity.

We observed oral manifestations of COVID-19 during infection (vesiculobullous lesions, acute gingivitis, palate ulcers, and sore throat) in 18% of patients, consistent with previous reports [24]. The patients were all healthy with no prior medical issues, but 19% reported smoking, which did not correlate with any breathing difficulties during the disease. Symptoms of SARS-CoV-2 infection reported by our participants included fever, chills, cough, shortness of breath, fatigue, muscle or body aches, headache, anosmia or ageusia, sore throat, congestion or runny nose, nausea or vomiting, diarrhea, and chest pain or pressure. Additionally, twelve reported ageusia, with four of these experiencing oral lesions.

Our study was limited by the lack of optimization of the salivary ELISA assays. The plates used were manufactured for serum testing; however, we tested saliva based on previously published studies as mentioned in the methods. We only included individuals with mild disease, so further analysis of assay characteristics is needed across the full spectrum of COVID-19 severity, not least because salivary IgA positivity may correlate with both disease duration and clinical status [8]. Furthermore, the population represented patients with a first episode of COVID-19, and they had not been vaccinated. There is a need for real-life studies examining the performance of an optimized assay in the present vaccinated and exposed population. Emphasizing the significance of these samples is essential, given that all were collected prior to the enforcement of the vaccine mandate. This offers a distinct opportunity for valuable future epidemiological investigations, facilitating a comparison of data before and after the mandate. Antibodies are key components of immune responses against novel viral infections such as SARS-CoV-2. Understanding their durability and their system compartmentalization across a diverse population is critical to monitoring seroprevalence in communities, selecting plasma donors for treatment, and designing vaccines.

## Conclusions

Here, we provide evidence that IgG and IgM/IgA responses to SARS-CoV-2 persist in the serum and are readily detectable in saliva. The detection of serum and salivary anti-SARS-CoV-2 antibodies using ELISA could provide a rapid and specific test for COVID-19. Further optimization of these salivary assays is now needed. Saliva may be a valuable diagnostic biofluid for both virus and antibody measurements. Since SARS-CoV-2 initially replicates in the oro- and nasopharyngeal tracts, it will be critical to further establish the nature and kinetics of salivary antibodies at the earliest time points post-infection in contact-traced individuals and their type to determine if there is a correlation between protective immunity and COVID-19 disease progression. There is now a need for further population-scale studies to optimize commercially available assays.
